# Transformative impact of three decades of nitrogen-based fertilization on diazotrophic communities and co-occurrence patterns in soils of Northeast China

**DOI:** 10.1128/spectrum.01443-24

**Published:** 2025-07-21

**Authors:** Yinghui Zhou, Lingzhi Liu, Bingqing Guo, Faryal Babar Baloch, Feng Wang, Yueshu Huang, Shuangyi Li, Tingting An, Bingxue Li, Jingkuan Wang

**Affiliations:** 1 College of Land and Environment, Shenyang Agricultural University 700623, Shenyang, China; 2 Key Laboratory of Arable Conservation in Northeast China, Ministry of Agriculture and Rural Affairs, College of Land and Environment, Shenyang Agricultural University 700623, Shenyang, China; Ruhr-Universitat Bochum, Bochum, Germany

**Keywords:** urea and organic fertilization, *nifH *gene, potential N_2_-fixation, diazotrophic community structure, co-occurrence network

## Abstract

Biological nitrogen (N) fixation (BNF) is a crucial biogeochemical process on Earth, in which diazotrophs play a significant role. The impact of long-term nitrogen (N) input from fertilization on diazotroph responses remains unclear. In this study, we investigated diazotrophic abundance, diversity, and potential N_2_-fixation activity in a 29-year maize (*Zea mays* L.) grown brown soil experiment with CK (unfertilized control), N4 (270 kg N ha^−1^ a^−1^ urea), and M2N2 (135 kg N ha^−1^ a^−1^ urea, 135 kg N ha^−1^ a^−1^ pig manure). We analyzed the *nifH* gene abundance and diversity using quantitative polymerase chain reaction (qPCR) and high-throughput sequencing, and determined the nitrogenase activity with the acetylene reduction method. Our results showed that N fertilization inhibited the soil’s potential N_2_-fixation activity compared to CK. Specifically, N4 had no impact on diazotrophic abundance and alpha diversity, while M2N2 increased both. Prolonged fertilization inhibited the phylum *Cyanobacteria*, *Nitrospirae*, and an unclassified phylum, while improving the phylum *Proteobacteria*. Additionally, N4 enhanced the phylum *Deinococcus-Thermus*. Monte Carlo test and canonical correlation analysis revealed that factors such as available N and pH played crucial roles in community construction. Network analysis indicated that fertilization enhanced diazotroph relationships, with N4 having the most complex network. The genera actively connected, or “common species,” differed in moderate abundance, contributing to diazotrophic community stability. Long-term fertilization reduced soil N_2_-fixation activity, prompting diazotrophs to form intricate co-occurrence networks in response to environmental shifts induced by nitrogen accumulation.

**IMPORTANCE:**

Fertilization has the potential to impact nitrogen (N) cycling within soil ecosystems. However, the response of diazotrophs to environmental changes induced by long-term N input remains largely unknown. Our research has uncovered that while inorganic N fertilizer did not significantly alter diazotrophic abundance and diversity, a combination of organic manure and mineral N fertilizer resulted in elevated levels of both. Moreover, the community structure of diazotrophs was found to be significantly influenced by nearly 30 years of N-based fertilizer application, with the key driving factors being the availability of N and soil pH. Network analysis revealed various co-occurrence patterns, indicating that fertilization led to increased interactions among diazotrophs, suggesting a more competitive dynamic as a result of prolonged N fertilization. We anticipate that these novel and significant findings will contribute to the development of more sustainable practices for N fertilizer utilization and enhance our understanding of soil diazotrophic diversity.

## INTRODUCTION

In response to the rising global population and the consequent demand for consumable and industrial resources, fertilization has become a crucial component of agricultural practices (1). In the past century, the role of chemical fertilizers has become increasingly essential, with nitrogen-based fertilizers particularly noted for significantly increasing crop yields beyond what natural conditions can achieve (2). Specifically, in China, the average annual nitrogen application over the last decade has been 217.53  kg N ha^−1^ year^−1^ (https://www.fao.org/faostat/en/#data/RFN). However, the heavy reliance on nitrogen-based fertilizers has led to adverse effects, including soil acidification, metal toxicity, heightened greenhouse gas emissions, and groundwater pollution, posing significant threats to soil quality, crop growth, biodiversity, and environmental health (3–5). Thus, the challenge of striking a delicate balance between fulfilling production needs and ensuring environmental protection is of utmost importance.

Fertilization significantly impacts soil microbial communities, which play crucial roles in soil biogeochemical cycling and ecological processes (6). The effects of long-term fertilization on soil microbial communities have garnered considerable attention. A 40-year experiment in Hertfordshire, United Kingdom, demonstrated that high levels of inorganic nitrogen fertilizers negatively affect bacterial richness and diversity, resulting in a less stable bacterial community structure over time. In contrast, organic amendments foster more stable bacterial communities (7). In the subarctic heath of northern Sweden, after 28 years of chronic inorganic nitrogen application, bacterial growth exhibited a pronounced response to fertilization, with higher growth rates observed in fertilized soils, while fungal growth remained unaffected (8). Additionally, organic fertilizers also influence soil bacterial community structure. In red clay soils of Southeast China, Nazir et al. (9) found that the succession of dominant bacterial species was significantly affected by various fertilization strategies, which included different proportions of inorganic and organic fertilizers. The relative abundance of *Acremonium* and *Mortierella* increased substantially with these strategies but decreased significantly when a high proportion of organic fertilizer was applied in a long-term study conducted from 1984 to 2013. Furthermore, a 25-year experiment in the North China Plain demonstrated that long-term fertilization regimes significantly altered soil bacterial community structure. Diversity was notably higher in treatments that utilized a double manure rate compared to mineral fertilizers (10). In summary, the long-term application of both organic and inorganic fertilizers distinctly shapes soil microbial communities, influencing their richness, diversity, and overall stability across various ecosystems.

Diazotrophic microorganisms play a critical role in biological nitrogen fixation (BNF), a crucial process for converting atmospheric nitrogen into an available anionic form in the soil. This natural process contributes up to 100 Tg N year^−1^ to terrestrial ecosystems (2, 11, 12), highlighting the need to explore more sustainable pathways for agricultural practices. The abundance and community structure of diazotrophs prove highly sensitive to soil physical and chemical properties (13). The *nifH* gene encodes the conserved subunit of the dinitrogenase iron protein in diazotrophs, serving as a common molecular marker for investigating their abundance and diversity across diverse environmental conditions (14, 15). The diversity of *nifH* is intricately linked to soil ecosystem functioning. The nitrogenase gene database, particularly the *nifH* gene, represents one of the largest non-ribosomal gene data sets from uncultivated microorganisms, making *nifH* widely applicable to diazotrophic studies (16). Furthermore, the effect of *nifH* in response to fertilization and subsequent changes in soil variables varies according to soil taxa (17), climates (18), types and rates of fertilizers applied (19), and application durations (20). Understanding these dynamics is crucial for comprehending the nuanced impact of fertilization on diazotrophic communities.

Multiple physical and chemical properties, such as pH, total nitrogen (TN), total carbon (TC), carbon-nitrogen ratio (C/N), available phosphorus (AP), ammonium nitrogen (NH_4_^+^–N), and nitrate nitrogen (NO_3_^−^–N), have been demonstrated to be influenced by artificial fertilization (21–23). Furthermore, changes in these soil variables often coincide with shifts in microbiota composition. The soil microbiota, a crucial component reflecting terrestrial ecosystem conditions, has recently garnered attention from researchers in agriculture, pedology, and microbiology (24–26). With the widespread adoption of genomic techniques, numerous studies have described bacterial abundance, diversity, and community structure in soil. Information on diazotroph distribution in both soil and aquatic environments has been acquired (26–29). For example, in Mediterranean rainfed cereal systems, the number of arbuscular mycorrhizal fungal spores and root colonization correlated with pH, AP, and C/N (30). Similarly, in forest soil, the bacterial community structure was influenced by TN and pH (31). Nevertheless, our understanding of how these nitrogen-fixing microorganisms respond to long-term fertilization remains limited. The complex and fascinating nature of soil, combined with the rapid advancements in agricultural engineering, contributes to the unknown effects of accumulated fertilization on diazotrophic bacteria.

In this study, we present the results of a 29-year experiment investigating the response of diazotrophs to the application of inorganic N-fertilizers (N4) and a combination of organic and inorganic N (M2N2) at different soil depths (0–20 cm and 20–40 cm beneath the surface). Our objective is to understand how nitrogen-fixing bacteria respond to extensive fertilization practices. The hypotheses guiding our research are as follows: (i) prolonged fertilization, regardless of the nitrogen source (inorganic or organic), is expected to reduce both the abundance and diversity of diazotrophs, leading to a decline in soil N_2_-fixation activity. (ii) Changes in the abundance, diversity, and community structure of diazotrophs are likely influenced by specific environmental factors, particularly the content of ionic nitrogen. (iii) With continued nitrogen application, dominant species are likely to be replaced by new ones. These “common species,” characterized by lower abundance or even rare occurrences, may become prominent members of the microbial community, undergoing shifts in response to fertilization.

## MATERIALS AND METHODS

### Soil sample collection

Soil samples were collected from the long-term fertilization experimental station, established in 1987 at Shenyang Agricultural University, located at coordinates 41°49′ N, 123°34′ E, in Liaoning province, China. Maize (*Zea mays* L.) is the predominant crop in this region. The soil type at the site is classified as Hapli-Udic Cambisol, according to the FAO soil classification system. This area experiences a continental monsoon climate, with an average annual temperature of 8.33°C ± 0.70°C and precipitation of 659.65 ± 206.34 mm, respectively, during 2011–2016.

The experimental design consisted of three distinct fertilization treatments applied across three plots, each measuring 69 m^2^. The fertilization system was detailed in Table 1: (i) CK—representing no fertilization; (ii) N4—involving the application of urea as an inorganic nitrogen fertilizer at a rate of 270 kg N ha^−1^ year^−1^; and (iii) M2N2—involving the combined use of urea and pig manure as organic nitrogen fertilizer, with both organic and inorganic fertilizers applied at an equivalent rate of 135 kg N ha^−1^ year^−1^ each. All fertilizers were uniformly applied as a basal fertilizer once before sowing in early May on an annual basis.

**TABLE 1:** Group names of the long-term fertilization system

Fertilization	Sampling depth (cm)	Group name
None fertilization	0–20	CK20
	20–40	CK40
Urea 270 kg N hm^−2^ a^−1^	0–20	N420
	20–40	N440
Urea 135 kg N hm^−2^ a^−1^ Pig manure 135 kg N hm^−2^ a^−1^	0–20	M2N220
	20–40	M2N240

On 5 November 2016, after the harvest, soil samples were obtained using the 5 cm diameter soil core method. In triplicate, samples were systematically collected from two distinct soil layers (0–20 cm and 20–40 cm) at each plot, resulting in a total of 18 samples representing six treatments: CK20, CK40, N420, N440, M2N220, and M2N240. For each sample, material from five randomly selected cores within a designated plot was combined. After removing gravel and roots, the composite sample was divided into two segments. One segment was immediately stored at −80°C for subsequent DNA extraction for molecular analysis, while the other segment underwent analysis to determine the physical and chemical properties of the soil, including the evaluation of key parameters relevant to soil quality and composition.

### Determination of fundamental soil properties

All physical and chemical properties were determined using established methods (32). The pH value was measured in a soil-water suspension with a ratio of 1:2.5 using a pH meter (Metrohm 702, Herisau, Switzerland). Ammonium-N (NH_4_^+^–N) and nitrate-N (NO_3_^−^−N) concentrations were extracted with 2 mol L^−1^ KCl and quantified through a continuous flow analyzer (Auto Analyzer 3, SEAL, Germany). AP was extracted with 0.5 mol L^−1^ NaHCO_3_ and determined using the Olsen method (33). TN and TC were assessed using an elemental analyzer (Elementar Vario EL III, Germany). A 35 mg dry soil sample was carefully packed into a 6 × 6 × 12 mm pressed tin boat (CN01031, CHNOS, China) and then placed into the automatic sampler according to the manufacturer’s guidelines.

### Measurement of N_2_-fixation activity

The N_2_-fixation activity was evaluated using the acetylene reduction method following the procedures outlined by Patra et al. (34) and Chen et al. (35). Operational adjustments were made to accommodate reaction containers. The primary procedures involved the precise addition of fresh soil equivalent to 10 g of oven-dried soil into a sterile 110 mL brown penicillin bottle. A 2 mL solution containing glucose (1 mg C g^−1^ dry soil) and disodium malate (1 mg C g^−1^ dry soil) was added to ensure sufficient carbon (C) availability. The bottle atmosphere was then replaced with a 9:1 mixture of air and pure C_2_H_2_ (99.99%). Subsequently, the airtight bottles were incubated in the dark at 25°C for 48 h. As diazotrophs have the capability to reduce acetylene (C_2_H_2_) to ethylene (C_2_H_4_), the soil’s potential N_2_-fixation activity was quantified based on the rate of C_2_H_4_ production (n mol C_2_H_4_ kg^−1^ soil h^−1^). The concentration of C_2_H_4_ was determined using gas chromatography with a flame ionization detector (Shimadzu GC-2010 plus, Japan).

### DNA extraction and quantitative polymerase chain reaction

Total DNA extraction from 0.5 g (fresh weight) soil samples was carried out using the Magnetic Soil and Stool DNA Kit (Tiangen, Beijing) in accordance with the manufacturer’s instructions. Concentrations were determined using the Nanodrop ND-2000 (Nanodrop Technologies; Thermo Scientific, US). Quantitative PCR (qPCR) was employed to assess *nifH* abundance using primer pairs (F: 5′-AAAGGYGGWATCGGYAARTCCACCAC-3′; R: 5′-TTGTTSGCSGCRTACATSGCCATCAT-3′) as described by Rösch et al. (36) and Feng et al. (37). Reactions were performed on the ABI Step One Plus Real-time PCR System (Applied Biosystems, US) following the manufacturer’s guidelines (38). The 20 µL reaction mixture consisted of 10 µL of Ultra SYBR mixture (CWBIO, China), 0.8 µL of each primer, 2 µL of template DNA, and 6.4 µL ddH_2_O to reach a final volume of 20 µL. Negative controls were implemented by substituting template DNA with ddH_2_O. The PCR conditions were as follows: 5 min at 94°C, followed by 45 cycles of 15 s at 95°C, 1 min at 52°C–60°C, and 1 min at 72°C. A melting curve analysis was conducted post-assay to assess the specificity of the PCR product. Each qPCR reaction was performed in triplicate. Amplification efficiencies for all detected genes were 90.79%, with an R^2^ of 0.980. Standard curves were generated using 10-fold serial dilutions of plasmids containing clone *nifH* genes.

### Sequencing and bioinformatic analysis

The amplification of the *nifH* gene was carried out using the previously mentioned primer pairs. PCR reactions were performed in a 30 µL volume, consisting of 15 µL of Phusion High-Fidelity PCR Master Mix (New England Biolabs), 0.2 µM of both forward and reverse primers, and approximately 10 ng of template DNA. The thermal cycling profile involved an initial denaturation step at 98°C for 10 min, followed by 30 cycles of denaturation at 95°C for 1 min, annealing at 55°C for 30 s, elongation at 72°C for 30 s, and a final extension at 72°C for 5 min. The resulting PCR products were mixed with an equal volume of 1× loading buffer (containing SYBR green) in equidensity ratios and subjected to electrophoresis on a 2% agarose gel for detection. Subsequently, purification was carried out using the GeneJETTM Gel Extraction Kit (Thermo Scientific).

The Ion Plus Fragment Library Kit 48 rxns (Thermo Scientific) was used to establish the libraries, following the manufacturer’s specifications. After quantification and examination using the LabChip GX Touch HT Nucleic Acid Analyzer (LabChip, PerkinElmer, US), sequencing was performed on the Ion S5 XL platform, producing 400 bp/600 bp single-end reads. The reads were compared with the Silva database (39) employing the UCHIME algorithm (40) to identify chimera sequences. Subsequently, the chimera sequences were systematically eliminated to obtain a clean reads data set. Sequence analysis was carried out using Uparse (v7.0.1001 [41]), with a similarity threshold set at ≥97% for assigning sequences to distinct operational taxonomic units (OTUs). Each representative sequence was taxonomically annotated using the Silva Database in conjunction with the Mothur algorithm. MUSCLE (Version 3.8.31 [42]) was utilized for multiple sequence alignment to explore phylogenetic relationships among different OTUs and to identify variations in dominant species across various samples or groups.

### Statistical analysis

Statistical analyses were performed using SPSS (Version 20.0 for Windows) and R software (Version 2.15.3). Variations in *nifH* gene copies and alpha diversity indexes among the fertilization treatments were evaluated through one-way analysis of variance (ANOVA), with Duncan’s test applied for multiple comparisons (*P* < 0.05). Additionally, a two-way ANOVA was employed to examine the interaction effects of fertilization treatments and sampling depth. Pearson’s correlation coefficients were utilized to explore relationships among *nifH* gene copies, alpha diversity indexes, potential N_2_-fixing activity, and soil properties. Furthermore, the Spearman correlation index was calculated to assess the correlations between species (the 30 most abundant genera) and environmental factors, and the analysis was performed using the Genescloud tools, a free online platform for data analysis (https://www.genescloud.cn).

Alpha diversity analysis, performed to assess species diversity complexity within a sample, utilized metrics such as the Shannon index, observed species, and goods-coverage. These metrics were computed using QIIME (Version 1.7.0). The Shannon index provides insights into both species richness and evenness, observed species quantifies the number of distinct species present, and goods-coverage evaluates the sequencing quality. To analyze community similarities and differences, an OTU-based hierarchical cluster analysis using the unweighted pair group method of arithmetic (UPGMA) means was conducted through the QIIME software. In order to identify the environmental factors that best explained the distinctions among diazotrophic communities, Monte Carlo permutation tests and canonical correlation analysis (CCA) were conducted using the vegan package, with the OmicShare tool (https://www.omicshare.com/tools/home/report/koenrich.html).

### Network construction

We focused on the top 100 genera based on their abundance to construct the network. Pearson correlation coefficient values (PCC) were calculated for each genus, resulting in a correlation coefficient matrix. Connections with a PCC less than 0.8 and a *P*-value exceeding 0.05 were excluded to filter out weakly related connections, and self-joining nodes were also eliminated. The network diagrams, depicting bacteria as nodes and values as edges based on the specified filtration criteria, were generated using Cytoscape (Version 3.8.2 [43]).

## RESULTS

### Effects of long-term fertilization on diazotrophic abundance, diversity, and nitrogenase activity

**Fig 1:**
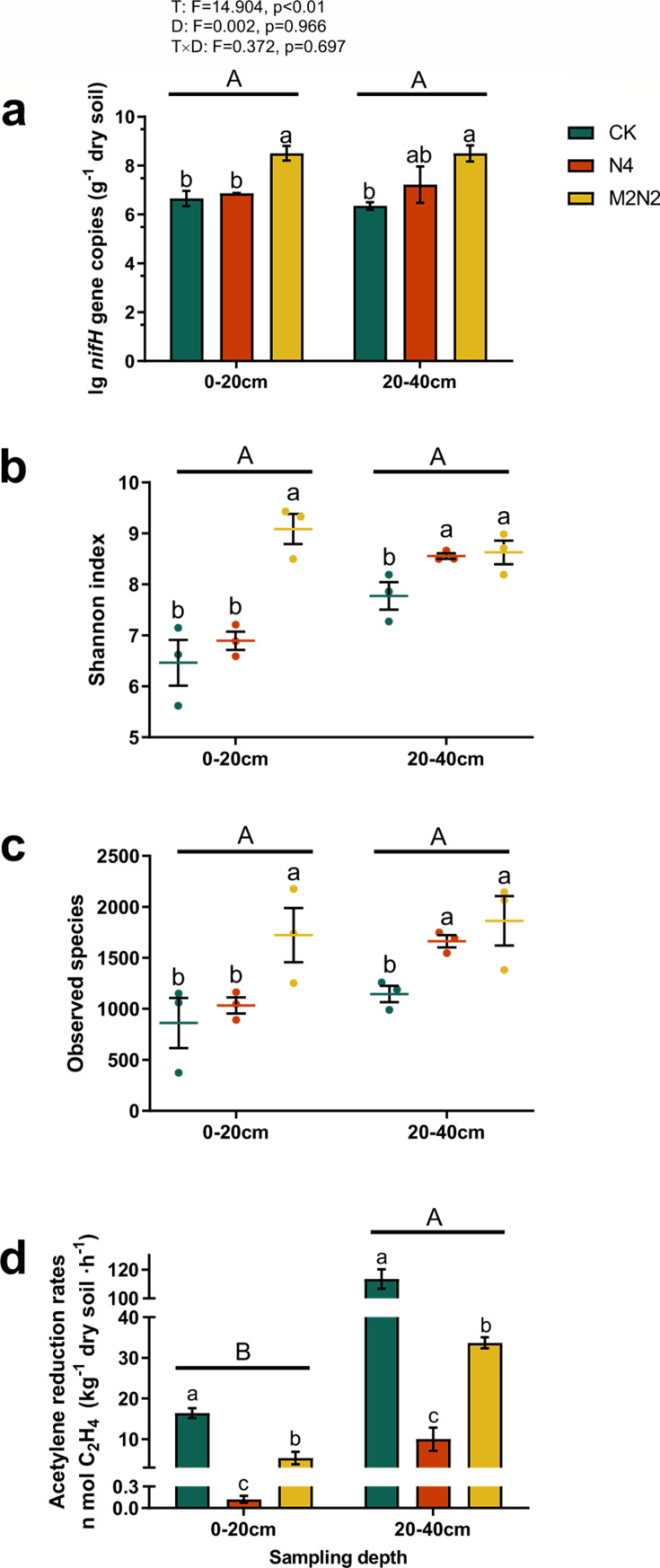
(**a**) *nifH* gene copies, alpha diversity based on (**b**) Shannon index, (**c**) observed species, and (**d**) potential N_2_-fixation activity in different long-term fertilization treatments and two sampling depths. Data are means ± SEM (*n* = 3). Colors of columns and dots represent fertilizing treatments according to the legend, as CK: none fertilization control; N4: urea as inorganic N fertilizer (270 kg N hm^−2^ a^−1^); and M2N2: urea combined with pig manure as organic N fertilizer (both equivalent to 135 kg N hm^−2^ a^−1^, respectively). Capital letters indicate significant differences at *P* < 0.05 between sampling depths (0–20 cm and 20–40 cm), and lowercase letters indicate significant differences at *P* < 0.05 among fertilizations according to Duncan’s test. “**a**” was analyzed by two-way ANOVA; T: fertilization treatments; D: sampling depth.

The abundance of the *nifH* gene ranged from 1.12 × 10^6^ to 1.17 × 10^9^ copies per gram of dry soil. Compared to the unfertilized control (CK), the application of both organic and inorganic fertilizers (M2N2) significantly increased *nifH* gene copies (*P* < 0.05), while the use of inorganic fertilizers alone (N4) did not exhibit significant differences (Fig. 1a). Overall, fertilization treatments had a significant impact on diazotrophic abundance (*P* < 0.01, Fig. 1a), while sampling depth did not significantly influence *nifH* gene abundance (*P* = 0.966). Additionally, no interaction effects between sampling depth and fertilization on diazotrophic abundance were observed (*P* = 0.697, Table S1).

After quality filtering and amino acid sequence screening, we obtained 1,874,913 high-quality sequences, with sample read numbers ranging from 52,085 to 126,011, depending on the soil samples (Table S2). These sequences were clustered into OTUs with at least 97% similarity for comparative analysis of diazotrophic community diversity across all soils, producing OTU numbers ranging from 28,000 to 83,710 (Table S2) and classified into 29 phyla, 68 classes, 128 orders, and 224 families. Long-term nitrogen (N) input significantly increased OTUs in comparison to the unfertilized control (CK) (Fig. S1). Two-way ANOVA results indicated a significant impact of fertilization on OTU numbers (*P* < 0.01), while sampling depth did not show a substantial influence (*P* = 0.424). To assess alpha diversity, the Shannon index and observed species were compared among different treatments, with goods-coverage used as a quality control measure for sequencing (Table S3). Overall, fertilization enhanced the alpha diversity of the *nifH* gene (Fig. 1b and c). Specifically, M2N2 significantly improved alpha diversity compared to CK (*P* < 0.05), whereas the N4 treatment did not exhibit notable differences. This pattern was more pronounced in the surface soil, echoing the gene abundance results.

The rates of ethylene production ranged from 0.12 to 113.43 n mol C_2_H_4_ kg^−1^ soil h^−1^ across the six treatments. Prolonged input of inorganic nitrogen (N4) notably reduced the potential N_2_-fixation activity of the soil. Interestingly, the combined use of organic fertilizer (M2N2) alleviated the inhibitory effect caused by mineral fertilization (Fig. 1d).

### Effects of soil properties on diazotrophic abundance, diversity, and nitrogenase activity

Pearson’s correlation analysis revealed that, across all soil samples, encompassing both surface and subsurface layers, *nifH* gene abundance exhibited a positive correlation with AP and nitrate nitrogen (NO_3_^−^–N) levels (*P* < 0.05, Table 2). Regarding alpha diversity, as represented by the Shannon index and observed species, there was a positive correlation with NO_3_^−^–N, but a negative correlation with the ammonium to nitrate ratio (NH_4_^+^/NO_3_^−^) (*P* < 0.05, Table 2). Furthermore, the number of OTUs displayed positive correlations with ammonium nitrogen (NH_4_^+^–N) and nitrate nitrogen (NO_3_^−^–N), while showing a negative correlation with pH (*P* < 0.05, Table S3). Additionally, Pearson’s correlation analysis indicated that N_2_-fixation activity was significantly and positively correlated with pH values, but significantly and negatively correlated with TC, the ammonium to nitrate ratio (NH_4_^+^/NO_3_^−^), and nitrogen content (TN, NH_4_^+^–N, and NO_3_^−^–N) (*P* < 0.05, Table 2).

**TABLE 2:** Pearson’s correlation coefficients between diazotrophic parameters and soil properties

	TN	TC	C/N	AP	NH_4_^+^–N	NO_3_^−^–N	NH_4_^+^/NO_3_^−^	pH
nifH gene copies	0.189	0.155	−0.283	**0.519^*a*^**	0.092	**0.534^*a*^**	−0.466	0.098
Significance (2-tailed)	0.453	0.539	0.256	0.027	0.718	0.022	0.051	0.699
Shannon index	−0.038	−0.087	−0.351	**0.544^*a*^**	0.081	**0.597^*b*^**	**−0.714^*c*^**	0.222
Significance (2-tailed)	0.88	0.732	0.153	0.02	0.751	0.009	0.001	0.376
Observed species	−0.082	−0.124	−0.313	0.431	0.198	**0.598^*b*^**	**−0.516** ^***a***^	0.158
Significance (2-tailed)	0.747	0.624	0.207	0.074	0.431	0.009	0.028	0.531
N_2_ fixation activity	**−0.579^*a*^**	**−0.542^*a*^**	0.39	−0.301	**−0.735^*c*^**	**−0.555^*a*^**	**−0.512^*a*^**	**0.558^*a*^**
Significance (2-tailed)	0.012	0.020	0.110	0.225	0.001	0.017	0.030	0.016

^*a*^ Bold value indicates correlation is significant at the 0.05 level (2-tailed).

^*b*^ Bold value indicates correlation is significant at the 0.01 level (2-tailed).

^*c*^ Bold value indicates correlation is significant at the 0.001 level (2-tailed).

In general, nitrate nitrogen (NO_3_^−^–N) content significantly impacted diazotrophic abundance, diversity, and soil N_2_-fixation activity. Similarly, the availability of phosphorus also influenced abundance and diversity. Notably, soil N_2_-fixation activity, the most sensitive parameter, could be affected by various environmental factors, including total carbon and nitrogen-related variables (TN, NH_4_^+^–N, NO_3_^−^–N, and NH_4_^+^/ NO_3_^−^) as well as pH value. Among these factors, NH_4_^+^–N emerged as the most influential one (Pearson’s coefficient = −0.735, *P* < 0.001), despite not correlating with diazotrophic abundance and diversity.

### Community structure analysis and its correlation with soil properties

**Fig 2:**
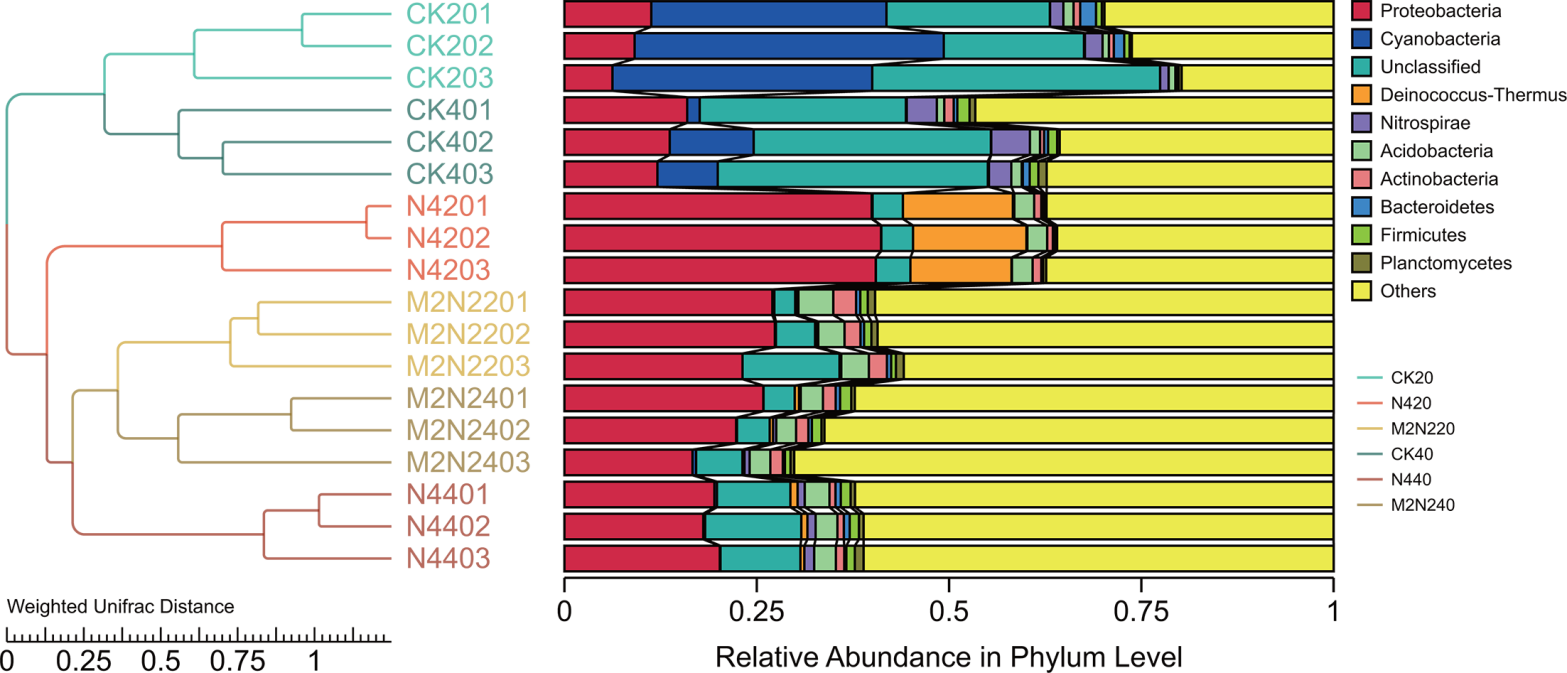
Illustrates the UPGMA cluster analysis, employing weighted Unifrac distance matrices for different fertilization treatments and sampling depths. The analysis was conducted on triplicate plots, and the group name was “treatment (corresponding to those in Fig. 1) plus sampling depth (20: 0–20 cm; 40: 20–40 cm) plus sample number.”

Assessing the similarities and differences in diazotrophic community compositions, we conducted a UPGMA cluster analysis based on OTU composition to gain a deeper understanding of species complexity. The communities formed two main clusters: unfertilized samples of CK20 and CK40, and fertilized samples of N4 and M2N2 (Fig. 2). Analyzing the dominant phyla (top 10 in relative abundance, Fig. 2), in the surface soil, fertilization inhibited the abundance of *Cyanobacteria*, *Nitrospirae*, and an unclassified phylum, while notably improving the abundance of the phylum *Proteobacteria*. Additionally, the N4 treatment significantly enhanced the abundance of *Deinococcus-Thermus*, which was rarely found in other samples. At the genus level, four genera of *Cyanobacteria* (*Cylindrospermum*, *Calothrix*, *Trichormus*, and *Anabaena*) were inhibited in both fertilized treatments (Fig. S2). While inorganic N input led to the blossoming of the genera *Granulicella*, *Thermus*, *Dyella*, *Rhodanobacter*, and mixed fertilization increased the genera *Gemmata*, an unclassified genus of *Acidobacteria*, *Ramlibacter*, *Rhodoplanes*, *Sideroxydans*, and *Pseudoxanthomonas*. In the subsurface soil, the trend of change at the phylum level was similar to the situation in the superficial soil. At the genus level, N4 treatment elevated *Paenibacillus*, *Rhodospirillum*, *Thioalkalivibrio*, and *Sphingopyxis*, and M2N2 treatment raised the genera *Croceicoccus*, *Bordetella*, and *Massilia*. The genera *Nitrospira*, *Methylocystis*, and *Granulibacter*, which were abundant in CK40, were notably inhibited after artificial N input (Fig. S2).

**Fig 3:**
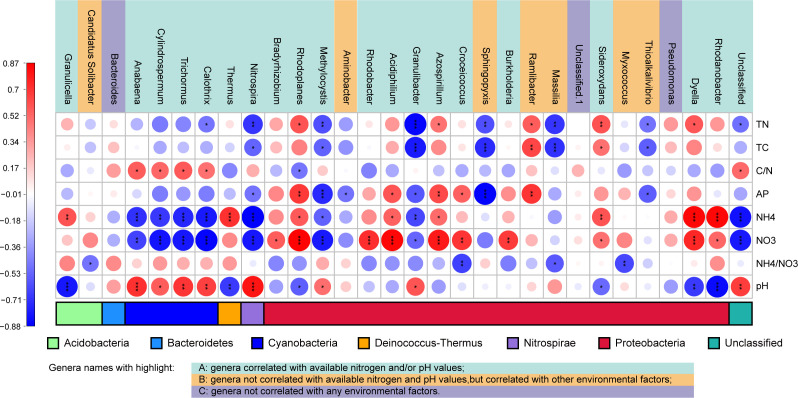
Heatmap illustrating the Spearman correlation between the abundance of the top 30 genera and environmental factors. Dot color and size represent Spearman correlation indexes, while the color of the bar at the bottom indicates the phylum (as per the legend). Asterisks denote significance (*: *P* < 0.05, **: *P* < 0.01, ***: *P* < 0.001).

The analysis included an examination of soil properties and the abundance of diazotrophic genera, accompanied by the calculation of Spearman correlation indexes (Table S6). Each environmental factor was associated with the abundance of specific genera, particularly those with high abundance (top 30). Notably, the most influential factors were two available nitrogen forms (NO_3_^−^–N, correlated with 18 genera; NH_4_^+^–N, correlated with 16 genera) and pH (significantly correlated with 14 genera) (Fig. 3).

The investigation of the genetic information of the most abundant 30 genera (Fig. S3) unveiled their origins from seven phyla: 20 genera from the *Proteobacteria* phylum, four from *Cyanobacteria*, two from *Acidobacteria*, and four from each of the phyla *Bacteroidetes*, *Deinococcus-Thermus*, *Nitrospirae*, and an unclassified phylum.

The 30 genera can be broadly categorized into three groups based on their relationships with environmental factors: (i) genera correlated with available nitrogen and/or pH values (genera names highlighted in cyan in Fig. 3), primarily distributed in the phyla *Cyanobacteria*, *Deinococcus-Thermus*, *Nitrospirae*, *Acidobacteria*, and *Proteobacteria*. (ii) Genera not correlated with available nitrogen and pH values but associated with other factors such as total nitrogen, total carbon, available phosphorus, and ammonium nitrate ratio (genera names highlighted in orange in Fig. 3), distributed in the phyla *Acidobacteria* and *Proteobacteria*. (iii) Three insensitive genera that were unrelated to all these environmental factors: the genus *Bacteroides* of the phylum *Bacteroidetes*, the genus *Pseudomonas*, and an unclassified genus of the phylum *Proteobacteria* (genus names highlighted in purple in Fig. 3).

**Fig 4:**
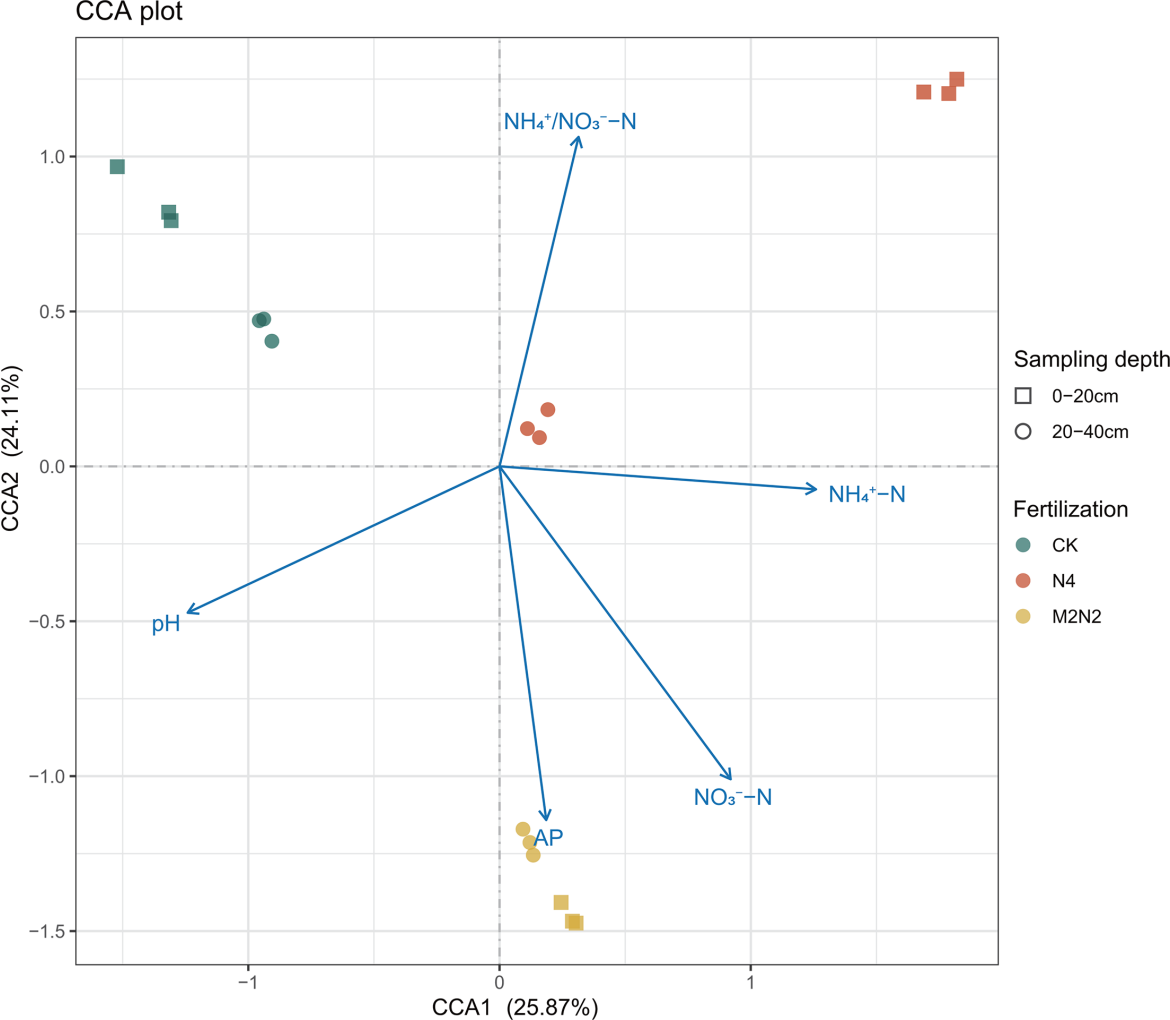
CCA depicting the diazotrophic community structure across all soil samples from the 29-year (1987–2016) long-term fertilization experiment. Arrows' positions and lengths signify the directions and strengths of environmental factors influencing diazotrophic communities.

A Monte Carlo test was conducted to analyze the relationships between environmental factors and diazotrophic community structure (Table S7). The results revealed that, with the exception of TC, TN, and the C/N ratio, the other five examined soil properties were closely correlated with diazotrophic community structure (*P* < 0.01). This underscores the sensitivity of nitrogen-fixing populations in responding to environmental changes, as reflected by the following ranking of correlating coefficients (*r* value): NO_3_^−^–N (*r* = 0.938, *P* = 0.001) > pH (*r* = 0.885, *P* = 0.001) > NH_4_^+^–N (*r* = 0.799, *P* = 0.001) > AP (*r* = 0.671, *P* = 0.003) > NH_4_^+^/NO_3_^−^ (*r* = 0.616, *P* = 0.002). Building upon this, selected soil properties and OTU data were further analyzed using CCA (Fig. 4). These soil variables accounted for 49.98% of the variation in community composition, with the first two axes explaining 25.87% and 24.11% of the variation, respectively. As shown in Fig. 4, N4 and M2N2 were clustered together and distributed at the positive end of CCA1, distinct from the unfertilized CK, indicating a significant shift due to long-term fertilization. According to the vectors, diazotrophic populations in CK were associated with higher pH values and the NH_4_^+^/NO_3_^−^ ratio, those in N4 were linked to higher ammonium content and the NH_4_^+^/NO_3_^−^ ratio, while populations in M2N2 treatments were positively correlated with available phosphorus. These soil properties also exhibited correlations with most of the abundant genera (Fig. 3; Table S6).

### Co-occurrence network analysis

**Fig 5:**
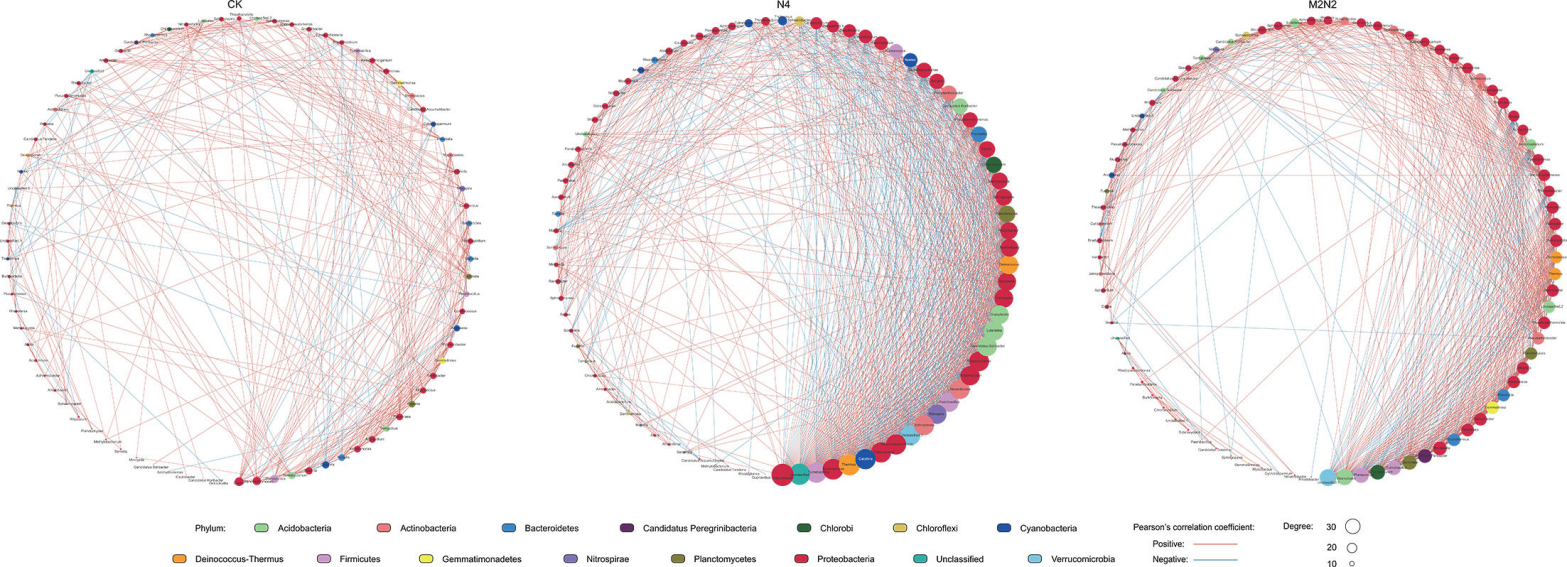
Co-occurrence networks at the genus level for three fertilization treatments. Node size represents the degree of connectivity, and node color indicates the phylum, as specified in the legends at the bottom. Red edges signify positive correlations, while blue edges indicate negative correlations (*P* < 0.05, Pearson correlation coefficient >0.8 or <−0.8). Treatment labels: CK (none fertilization control), N4 (urea as inorganic N fertilizer, 270 kg N hm^−2^ a^−1^), M2N2 (urea combined with pig manure as organic N fertilizer, both equivalent to 135 kg N hm^−2^ a^−1^, respectively).

To explore the impact of fertilization on the co-occurrence patterns within the diazotrophic community, three networks were constructed based on genus data for different fertilizing treatments (CK, N4, and M2N2). Figure 5 provides insights into the relationships among diazotrophs in each treatment, reflecting credible abundance calculations. Red edges in the figure denote positive correlations, while blue ones indicate negative Pearson’s correlations (*P* < 0.05, PCC cutoff value 0.8). Node size represents the connecting degree of the annotated genus, and the color indicates the phylum. This analysis encompassed 96 genera from 15 phyla in the bacterial kingdom. The CK network included 82 nodes, representing the interactions among 82 genera in CK soil. The N4 network featured 84 nodes, and the M2N2 network exhibited the maximum nodes, reaching 86. The CK network had only 346 edges, whereas the N4 network boasted 902 edges, and the M2N2 network had 659 edges. In terms of relational complexity, the number of edges in the N4 network was significantly higher than in the other two fertilizing treatments (Fig. 5).

We noted that an unclassified genus of the phylum *Verrucomicrobia* showed strong connections with other genera in the two fertilized networks, with a degree of 39 in N4 and 33 in M2N2, indicating its prominence as a “common species” in both treatments. Conversely, in the CK network, this genus did not demonstrate these characteristics, with a degree of 7. Additionally, two other notable genera, *Thermus* and *Deinococcus*, members of the aforementioned phylum *Deinococcus-Thermus* and abundant in N420, exhibited close relationships with many other genera.

## DISCUSSION

### Effects of long-term N-based fertilization on potential N_2_-fixation activity, diazotroph abundance, and diversity

Long-term nitrogenous fertilizer application has consistently been associated with a notable reduction in nitrogenase activity (44, 45). Liao et al. (46) documented a decline in N_2_-fixing activity with two application rates (50 and 100 kg N ha^−1^ year^−1^) of chemical nitrogen fertilizer, finding that the addition of phosphorus (P)-based fertilizer contributed to the recovery of activity. Additionally, in a distinct trial encompassing 33 years of wheat-maize rotation, the potential N_2_-fixing activity did not change in the N+P+potassium (K) treatment compared with the non-fertilized control, while the potential N_2_-fixing activity in the organic treatment exceeded that of CK and NPK treatments (47). In our study, nitrogen accumulation spanning almost three decades significantly repressed soil nitrogenase activity, with the most pronounced impact observed in the inorganic nitrogen input treatment. Interestingly, the supplementation of organic manure alleviated the reduction in activity compared to N4, suggesting that the incorporation of other elements, such as phosphorus, has the potential to mitigate the negative effects related to high-level nitrogen input. These findings highlight the potential of a fertilization approach combining organic and inorganic fertilizers for future consideration.

Diazotrophic microbiomes have long been recognized as vital components of agricultural ecosystems (48). With the advancements in molecular biological approaches, like qPCR (49, 50), as well as in metagenomics and metatranscriptomics (51–53), there is increasing interest in understanding how these nitrogen fixers respond to artificial nitrogen (N) input, an integral aspect of modern agriculture. Long-term fertilization has frequently been linked to a decrease in general soil microbial abundance (7, 54). In terms of diazotrophic abundance, acidic soil conditions can inhibit the growth and activity of nitrogen-fixing microbial populations, leading to a decrease in abundance (45). Some studies implied that high nitrogen content could inhibit diazotrophic abundance (55, 56). Furthermore, the interaction between these two factors may produce combined effects on diazotrophic abundance (35). Additionally, some ammonium-based fertilizers can release toxic levels of ammonium ions into the soil. High levels of ammonium can be detrimental to many soil microbes, leading to a decline in diazotrophic abundance (57). However, our study contradicts this perspective. Over nearly three decades of inorganic nitrogen application (N4 treatment), we observed no decrease in the abundance of the *nifH* gene compared with CK. Moreover, the application of combined inorganic and organic fertilizers significantly increased the gene copies (Fig. 1a). This observation aligns with the results of Shi et al. (58), where the application of inorganic plus organic manure for 41 years alleviated the decline in *nifH* abundance caused by inorganic fertilizer in upland red soil in Yunnan, China. Another study reported that a 4-year straw mulch promoted *nifH* copies in wheat-grown alkaline purple soil in Sichuan, China (59).

Several studies have suggested that the application of fertilizers may lead to a reduction in soil diazotrophic diversity. Long-term fertilization experiments, such as a 25-year inorganic nitrogen input study, indicated a significant decrease in OTU richness in acidic soil in Hunan, China (60). Similarly, shorter-term experiments, like a 5-year application of NH_4_NO_3_, resulted in a decline in *nifH* gene copies and OTU numbers in a fir plantation in Jiangxi, China (45). In contrast, our study does not support these conclusions. Chemical fertilizer, when compared to the control (CK), did not alter the Shannon indices and observed species (Fig. 1b and c). However, the compound fertilizer M2N2 significantly increased both alpha diversity indices (*P* < 0.05, Fig. 1b and c). Chen et al. (59) observed a similar trend, reporting that higher nitrogen fertilizer input with straw mulching enhanced observed species in the wheat rhizosphere. This aligns with our findings using organic manure combined with inorganic chemical fertilizer. Another study based on a 19-year site with a double rice-cropping system reported a significant improvement in the Ace index and Chao 1 index in a paddy field in Jiangxi, China (19). Overall, the number of abundant species in the M2N2 treatment was larger than in CK and N4 (Fig. S2). The diversity data remained consistent with the result that fertilizing treatments significantly improved alpha diversity at both sampling depths (Fig. 1b and c).

### Effects of environmental factors on diazotrophs

Pearson’s correlation analysis revealed the impact of environmental factors on diazotrophic abundance and diversity. Surprisingly, the nitrate nitrogen content exhibited a positive correlation with *nifH* abundance and diversity (Table 2). This finding contradicts our initial hypothesis. Previous studies (35, 56) suggested that prolonged N-based fertilization, leading to significant nitrogen accumulation, typically inhibits diazotrophic abundance and diversity. The unexpected result may be attributed to the unique community structure in this specific natural environment. The Spearman correlation results (Fig. 3) highlight that different genera respond diversely to environmental changes: some exhibit opposite reactions, while others appear impervious to these alterations. The varying abundance and structure of these genera might represent a strategic adaptation to cope with shifting environmental conditions.

The phosphorus factor (AP) exhibited a significant correlation with the abundance and alpha diversity of the nitrogen-fixing gene. Phosphorus (P) availability, often bound to highly weathered soil minerals and organic matter, frequently limits BNF (45). This constraint could be attributed to the biosynthesis of ATP and DNA, both crucial for duplication (61). Higher phosphorus availability may provide microorganisms with the opportunity to thrive and reproduce, potentially explaining the observed positive correlations between AP and diazotrophic abundance and diversity. However, the specific mechanisms involved require further discussion. The NH_4_^+^/NO_3_^−^ ratio demonstrated a negative correlation with *nifH* gene alpha diversity (Table 2). Li et al. (62) suggested that an optimal NH_4_^+^/NO_3_^−^ ratio enhances nodulation indices and nitrogenase activity in soybean rhizobia, thereby improving nitrogen-fixation efficiency. Nonetheless, our long-term fertilization experiment sites present a different scenario. After nearly three decades of N input, nitrogen accumulation might have surpassed what is considered an “optimal concentration.”

The potential N_2_-fixation activity demonstrated a negative correlation with TN, TC, NH_4_^+^–N, NO_3_^−^–N, and NH_4_^+^/NO_3_^−^ ratio, while exhibiting a positive association with pH value, as expected. Prolonged N input resulting in increased ionic N content can lead to soil acidification (63, 64), subsequently causing a significant reduction in nitrogenase activity, a well-documented phenomenon (44, 45). For nitrogen fixers, the process of fixing atmospheric nitrogen is energetically demanding (65, 66). In an environment with abundant or excessive available nitrogen, the energetic cost appears prohibitively high for generating additional nitrogenous components instead of prioritizing reproduction.

The environmental factors mentioned in this section demonstrated significant associations with the diazotrophic community structure, as indicated by the Monte Carlo test and CCA results (Fig. 4; Table S7). pH played a pivotal role in shaping the soil diazotrophic community (67). In addition to pH, N and P content were also linked to the distribution of dominant N fixers, as evidenced in the forest farm with *Casuarina equisetifolia* cultivation in Fujian, China (68). As microorganisms involved in nitrogen fixation, diazotrophs are likely to be associated with N content, including available N and total N (69). Ramirez et al. (70) found that bacterial communities across gradients of NH_4_NO_3_ are more structured by N and/or soil carbon availability than by shifts in soil pH associated with elevated N inputs. However, we did not observe significant correlations with TN, TC, and C/N. Furthermore, the undeniable impact of pH and available N did not differ significantly (Fig. 4).

### Crucial species in different fertilizing treatments

It is widely acknowledged that long-term fertilization can induce changes in the microbial community composition in soil (71–74). As expected, our study revealed alterations in the diazotrophic structure due to long-term N-based fertilization. While the abundance of diazotrophs was not reduced by N-based fertilizers, fertilization significantly impacted diversity and structure. In comparison to unfertilized treatments, N-fertilization suppressed the growth of certain phyla, such as *Cyanobacteria* and *Nitrospirae*, while promoting other diazotrophs (Fig. 2; Fig. S2). The phylum *Deinococcus-Thermus* exhibited notable abundance in the superficial soil of the N4 treatment (Fig. 2), constituting more than 14% of the entire community in N420 but being scarcely observed in other samples. *Deinococcus-Thermus*, first described in 1989, possesses an unusually high G+C composition (75) and is typically found in extremely dry and hot environments (76), indicating substantial biodiversity (77) in some instances. In network analysis, we also observed its close association with other diazotrophs. Notably, previous research mentioned the disappearance of certain extremophilic bacterial groups, including *Deinococcus-Thermus*, from soil after agricultural use (78). The persistence of this phylum in our farmland trial and its emergence as an abundant species after nearly 30 years of cultivation and fertilization pose an intriguing question for further exploration.

Co-occurrence networks provide valuable insights into potential interactions within microbial communities (47, 79). Xue et al. (80) observed a higher correlation in the soil fungal community following organic amendment, while Chen et al. (81) identified stronger commensal and mutual assemblage in the peanut rhizosphere network of phosphorus-correlated genera after 27 years of fertilization. In our investigation focusing on N_2_-fixing bacteria, the co-occurrence network suggested that artificial nitrogen input altered the co-occurrence pattern of diazotrophs. Within the N4 network, 312 out of 902 interactions exhibited negative PCCs, accounting for 34.59% of the total (in CK: 14.74%; M2N2: 21.24%). It became the most complex network, and the M2N2 network was also more intricate than the CK network. Fertilization appeared to enhance the connections among diazotrophs, with the most pronounced effect observed in the N4 treatment, indicating a more competitive environment for individuals. This could represent a strategy to maintain community stability and balance (82).

When discussing crucial or dominant species, the focus is often on species with relatively high abundance, typically no less than 1% (83, 84). Recently, some researchers have argued that rare species, usually comprising less than 0.1%, also play significant roles (85, 86). Other studies emphasize the importance of considering both aspects (87). In this study, our attention was directed toward species closely connected with others. Based on the network diagram and relevant data, the most active genera were not necessarily the most abundant or the rarest ones, as observed with *Bosea* in CK, *Granulicella* in N4, and the unclassified genus of the *Verrucomicrobia* phylum in M2N2. Although these genera typically constituted less than 1% of the total population, they demonstrated strong interactions with other members. This intriguing phenomenon is not only interesting but also inspiring. We have traditionally focused on the extremes in abundance, overlooking the “more common ones” that might also contribute to, or even sustain, the stability of the nitrogen-fixing community connections. The prevalence of these “common species” warrants further exploration in future studies.

### Conclusions

Long-term fertilization significantly reduced the soil potential for N_2_ fixation activity, but it did not result in a decline in diazotrophic abundance and alpha diversity. Over nearly 30 years of nitrogen input, diazotrophs formed more intricate co-occurrence networks as a response to intensified competition and adaptation to environmental shifts caused by changes in soil variables, such as available nitrogen content and pH values. Beyond the most abundant or the rarest populations, the “common species” might play a crucial role in a community and should be examined more thoroughly in the future, as exemplified by the genus *Granulicella* from the phylum *Acidobacteria* and the unclassified genus of the phylum *Verrucomicrobia*.

## Data Availability

The raw sequencing data supporting the findings of this study have been deposited in the NCBI Sequence Read Archive (SRA) under the BioProject accession number PRJNA1286943.

## SUPPLEMENTAL MATERIAL

Supplemental material spectrum.01443-24-s0001.pdf Table S1 to S7, Fig. S1 to S3, and legends.

ASM does not own the copyrights to Supplemental Material that may be linked to, or accessed through, an article. The authors have granted ASM a non-exclusive, world-wide license to publish the Supplemental Material files. Please contact the corresponding author directly for reuse.
