# Supplementary material for: Transformative impact of three decades of nitrogen-based fertilization on diazotrophic communities and co-occurrence patterns in soils of Northeast China
Source: Microbiol Spectr. 2025 Jul 21;13(9):e01443-24. doi: 10.1128/spectrum.01443-24 (PMC12403873; doi:10.1128/spectrum.01443-24)
Supplement: Supplemental material — Table S1 to S7, Fig. S1 to S3, and legends. [file spectrum.01443-24-s0001.pdf]

Transformative Impact of Three Decades of Nitrogen-Based Fertilization on Diazotrophic Communities and Co-occurrence Patterns in Soils of North-East China

Yinghui Zhou[1, 2], Lingzhi Liu[1, 2]*, Bingqing Guo[1, 2], Faryal Babar Baloch[1, 2], Feng Wang[1, 2], Yueshu Huang[1, 2], Shuangyi Li[1, 2], Tingting An[1, 2], Bingxue Li[1, 2]*, Jingkuan Wang[1, 2]*

1 College of Land and Environment, Shenyang Agricultural University, Shenyang, China

2 Key Laboratory of Arable Conservation in Northeast China, Ministry of Agriculture and Rural Affairs, College of Land and Environment, Shenyang Agricultural University, Shenyang, China

*Corresponding Author: Lingzhi Liu, E-mail: liulingzhi2006@syau.edu.cn; Bingxue Li, E-mail: libingxue@syau.edu.cn; Jingkuan Wang, E-mail: jkwang@syau.edu.cn

Yinghui Zhou: polarchow37@stu.syau.edu.cn (ORCID: 0009-0003-3143-6333)

Bingqing Guo: 1156079993@qq.com

Faryal Babar Baloch: faryalbabarbaloch@gmail.com

Feng Wang: 18809865828@163.com

Yueshu Huang: huangyueshu@stu.syau.edu.cn

Shuangyi Li: shy_li@syau.edu.cn

Tingting An: atting@syau.edu.cn

Key words: Urea and organic fertilization; *nifH* gene; Potential N_2-fixation; Diazotrophic community structure; Co-occurrence network

Table S1 Two-way ANOVA analysis: Impact of fertilization treatments (T) and sampling depth (D) on nifH gene abundance (lg) and OTU numbers

Factors	*nifH* gene abundance (lg)		OTU numbers	
	F	*p*	F	*p*
T	14.904	**<0.01**	10.424	**<0.01**
D	0.002	0.966	0.199	0.663
T×D	0.372	0.697	0.923	0.424

Table S2 Summary of usable sequences, post-quality filtering and screening of amino acid sequences and OTUs across all samples

Sample	Sequences	OTUs	Sample	Sequences	OTUs
CK20.1	52085	28000	CK40.1	75804	44447
CK20.2	99732	59591	CK40.2	55552	29481
CK20.3	77066	53429	CK40.3	115417	74201
N420.1	120156	83559	N440.1	120097	75134
N420.2	120044	83710	N440.2	126011	77466
N420.3	120045	81485	N440.3	120056	75245
M2N220.1	96600	60003	M2N240.1	120147	76339
M2N220.2	120169	77529	M2N240.2	120052	78810
M2N220.3	95804	58830	M2N240.3	120076	78375

CK: control; N4: inorganic nitrogen fertilization (270kg N hm^{-2} a^{-1}); M2N2: organic and inorganic fertilization (both equivalent to 135kg N hm^{-2} a^{-1} respectively).

Table S3 alpha diversity indices across all treatments.

	0-20cm			20-40cm		
	CK	N4	M2N2	CK	N4	M2N2
Shannon	6.643±0.449	6.895±0.179	9.085±0.296	7.773±0.269	8.556±0.055	8.628±0.234
observed species	862±246	1034±78	1723±267	1146±80	1664±61	1864±242
goods coverage	0.9957±0.0015	0.9933±0.0012	0.9913±0.0022	0.9920±0.0010	0.9887±0.0003	0.9867±0.0023

Table S4 Pearson's correlation coefficients between different soil physicochemical variables and OTU numbers

	TN	TC	C/N ratio	AP	NH_4^+-N	NO_3^--N	NH_4^+/NO_3^-	pH
OTU number	-0.003	-0.026	-0.119	0.058	**0.649****	**0.556***	0.076	**-0.481***

*p<0.05, **p<0.01.

Table S5 Soil properties in surface soil (0-20cm) and subsurface soil (20-40cm) of maize from a 29-year long-term fertilization experiment

Treatment	TN±SD	TC±SD	C/N±SD	AP±SD	NH_4^+-N±SD	NO_3^--N±SD	NH_4^+/NO_3^-	pH±SD
CK20	**0.98±0.02c****	**10.04±0.20b****	10.28±0.05a	**33.47±1.40b****	**1.27±0.07c****	**5.60±0.23c****	**0.227±0.003a****	5.85±0.07a
CK40	**0.63±0.04b**	**6.49±0.30b**	10.27±0.86a	**15.52±0.85b**	**0.52±0.01b**	**4.77±0.01c**	**0.109±0.002b**	5.99±0.13a
N420	**1.01±0.01b****	**10.01±0.07b****	9.91±0.03b	**26.60±1.14c****	**3.91±0.68a***	**16.46±0.35b****	**0.237±0.036a***	**4.38±0.03c****
N440	**0.63±0.03b**	**6.22±0.08b**	9.89±0.44a	**7.31±0.71c**	**2.08±0.14a**	**12.43±0.35b**	**0.167±0.009a**	**5.54±0.30b**
M2N220	**1.26±0.01a****	**12.51±0.02a****	9.95±0.04b	**188.42±0.51a****	2.91±0.52b	**24.08±0.16a****	0.121±0.021b	5.46±0.13b
M2N240	**0.74±0.04a**	**7.35±0.41a**	9.89±0.26a	**83.50±1.61a**	2.07±0.15a	**20.11±0.32a**	0.103±0.006b	5.76±0.14ab

The data represent means ± standard deviation (N=3). Different letters denote significant differences among fertilizer treatments in either surface or subsurface soil at p<0.05. Bold asterisks indicate significant differences between the two sampling depths, where: *p<0.05, **p<0.01. Treatment labels: CK (control), N4 (inorganic nitrogen fertilization, 270 kg N hm^{-2} a^{-1}), M2N2 (organic and inorganic fertilization, both equivalent to 135 kg N hm^{-2} a^{-1}, respectively)

Table S6 Spearman's correlation coefficients between soil physicochemical variables and the relative abundances of the top 30 diazotrophic genera

Genus name	TN	TC	C/N	AP	NH_4^+-N	NO_3^--N	NH_4^+/ NO_3^-	pH
Unclassified	**-0.487**	-0.280	**0.515**	-0.307	**-0.815**	**-0.794**	-0.067	**0.666**
Rhodanobacter	0.357	0.120	-0.338	-0.086	**0.851**	**0.527**	0.383	**-0.845**
Dyella	**0.575**	0.416	-0.292	0.364	**0.856**	**0.748**	0.067	**-0.672**
Pseudomonas	0.347	0.235	-0.152	0.137	0.290	0.290	-0.013	-0.337
Thioalkalivibrio	**-0.480**	**-0.549**	-0.199	**-0.559**	-0.017	-0.101	-0.162	-0.074
Myxococcus	-0.082	-0.142	-0.352	0.261	0.023	0.423	**-0.634**	0.118
Sideroxydans	**0.592**	**0.487**	-0.087	0.360	**0.597**	**0.491**	0.035	**-0.515**
Unclassified.1	0.001	0.043	0.261	0.062	0.066	0.109	0.030	-0.077
Massilia	**-0.690**	**-0.709**	-0.109	-0.300	-0.339	-0.153	**-0.569**	0.360
Ramlibacter	**0.559**	**0.644**	0.212	**0.694**	-0.026	0.252	-0.216	0.141
Burkholderia	0.083	0.028	-0.307	0.381	0.259	**0.672**	-0.443	-0.051
Sphingopyxis	**-0.604**	**-0.731**	-0.240	**-0.874**	-0.113	-0.451	0.164	-0.059
Croceicoccus	0.103	0.090	-0.290	**0.548**	0.231	**0.692**	**-0.659**	-0.112
Azospirillum	**0.483**	0.405	-0.215	**0.622**	**0.512**	**0.793**	-0.264	-0.339
Granulibacter	**-0.859**	**-0.793**	0.040	**-0.583**	**-0.635**	**-0.523**	-0.404	**0.587**
Acidiphilium	0.354	0.286	-0.298	**0.589**	**0.543**	**0.852**	-0.372	-0.293
Rhodobacter	0.083	-0.024	-0.428	0.305	0.395	**0.721**	-0.414	-0.216
Aminobacter	-0.337	-0.379	-0.020	**-0.481**	-0.298	-0.389	0.155	0.184
Methylocystis	**-0.607**	**-0.548**	0.179	**-0.734**	**-0.547**	**-0.793**	0.285	**0.496**
Rhodoplanes	**0.588**	0.438	**-0.471**	**0.681**	**0.573**	**0.871**	-0.378	**-0.552**
Bradyrhizobium	0.305	0.239	-0.245	0.414	0.394	**0.562**	-0.420	-0.335
Nitrospira	**-0.690**	**-0.531**	0.280	**-0.482**	**-0.884**	**-0.804**	-0.069	**0.812**
Thermus	0.106	-0.151	-0.411	-0.236	**0.713**	0.404	0.349	**-0.674**

Genus name	TN	TC	C/N	AP	NH_4^+-N	NO_3^--N	NH_4^+/ NO_3^-	pH
Calothrix	**-0.474**	-0.278	**0.493**	-0.444	**-0.788**	**-0.865**	0.268	**0.656**
Trichormus	-0.419	-0.223	**0.569**	-0.354	**-0.743**	**-0.804**	0.274	**0.697**
Cylindrospermum	-0.459	-0.295	**0.527**	-0.449	**-0.686**	**-0.807**	0.349	**0.559**
Anabaena	-0.260	-0.029	**0.561**	-0.140	**-0.711**	**-0.647**	0.155	**0.712**
Bacteroides	0.106	0.214	0.342	0.070	-0.274	-0.274	0.406	0.334
Candidatus Solibacter	-0.187	-0.214	-0.076	0.029	0.158	0.409	**-0.485**	-0.123

Values in bold indicate significant correlations at $p < 0.05$ or $p < 0.01$

Table S7 The correlations (r) and significance (p) were determined by Monte Carlo tests between the community structure and soil physicochemical environmental variables

Variable	r	p
TN	0.11926557	0.369
TC	0.08222862	0.495
C/N ratio	0.18638927	0.209
AP	**0.67148887**	**0.003**
NH_4^+-N	**0.79938149**	**0.001**
NO_3^--N	**0.93827407**	**0.001**
NH_4^+/ NO_3^-	**0.61623376**	**0.002**
pH	**0.88460248**	**0.001**

Values in bold indicate significant correlations at $p < 0.01$.

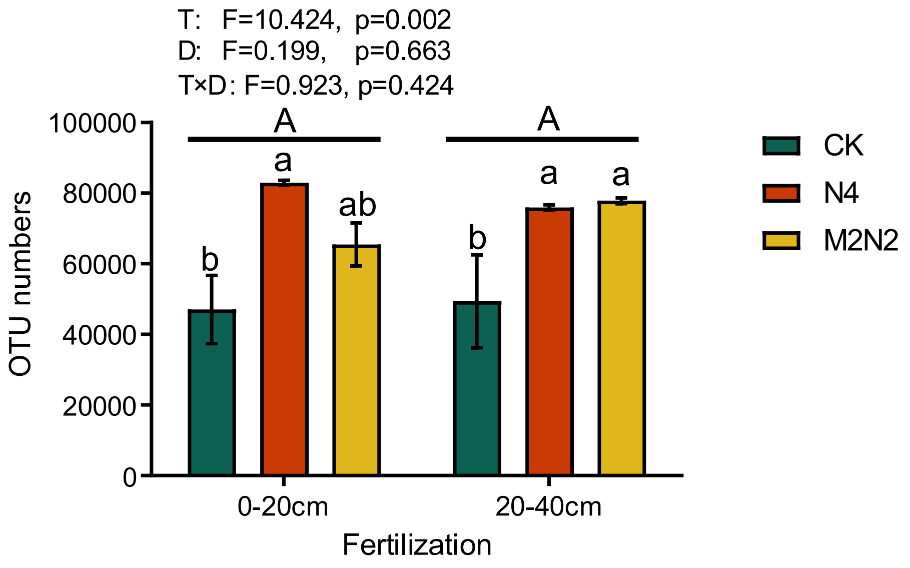

Fig. S1 Displays OTU numbers under various long-term fertilization treatments and two sampling depths. Data are presented as means ± SEM (n = 3). Treatment labels include CK (control), N4 (inorganic nitrogen fertilization, 270 kg N hm^{-2} a^{-1}), M2N2 (organic and inorganic fertilization, both equivalent to 135 kg N hm^{-2} a^{-1}, respectively). Statistical analyses were conducted using two-way ANOVA, with T representing fertilization treatments and D representing sampling depth

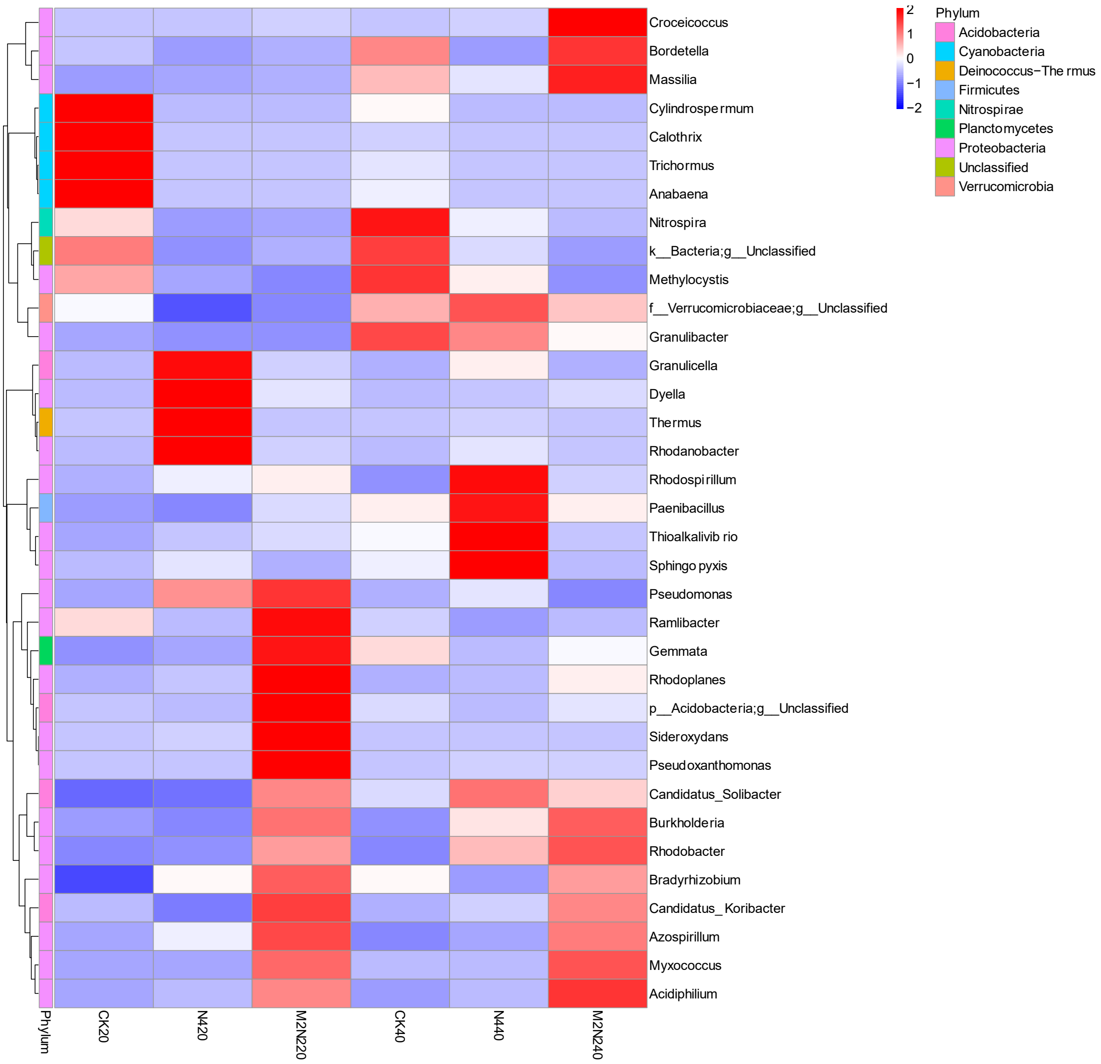

Fig. S2 Heatmap depicting diazotrophic community composition at the genus level under various treatments. Left-side colors represent phyla, and interior cubes indicate relative genus abundance. Treatments: CK (control), N4 (inorganic nitrogen fertilization, 270 kg N hm^{-2} a^{-1}), M2N2 (organic and inorganic fertilization, 135 kg N hm^{-2} a^{-1} each)

Tree plot

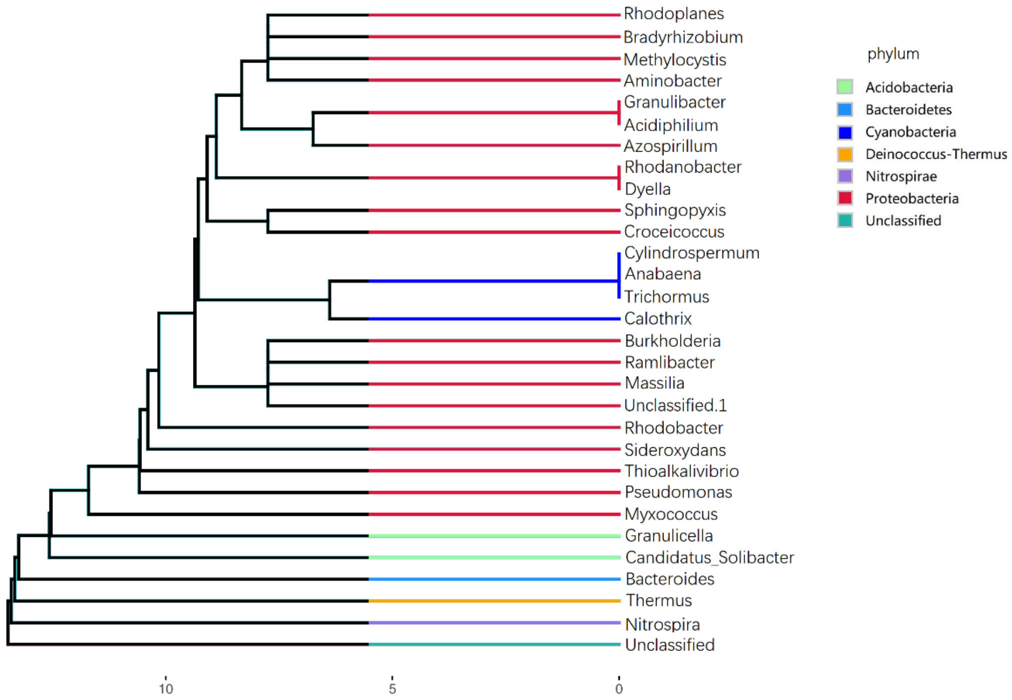

Fig. S3 A tree plot depicts the genetic information of the top 30 abundant genera. Branch colors indicate the respective phylum of each genus, as detailed in the legend on the right
